# Zeo-1, a computational data set of zeolite structures

**DOI:** 10.1038/s41597-022-01160-5

**Published:** 2022-02-22

**Authors:** Leonid Komissarov, Toon Verstraelen

**Affiliations:** Center for Molecular Modeling (CMM), Ghent University, Technologiepark-Zwijnaarde 46, B-9052 Ghent, Belgium

**Keywords:** Computational chemistry, Atomistic models, Density functional theory

## Abstract

Fast, empirical potentials are gaining increased popularity in the computational fields of materials science, physics and chemistry. With it, there is a rising demand for high-quality reference data for the training and validation of such models. In contrast to research that is mainly focused on small organic molecules, this work presents a data set of geometry-optimized bulk phase zeolite structures. Covering a majority of framework types from the Database of Zeolite Structures, this set includes over thirty thousand geometries. Calculated properties include system energies, nuclear gradients and stress tensors at each point, making the data suitable for model development, validation or referencing applications focused on periodic silica systems.

## Abstract (summary)

**Table:** 

Measurement(s)	potential energy
Technology Type(s)	Computational Chemistry
Factor Type(s)	Crystal structure, composition and topology

Machine-accessible metadata file describing the reported data: 10.6084/m9.figshare.17313236

## Background & Summary

Atomistic models are an essential tool for the prediction of thermodynamic, mechanical or biochemical properties of a substance. More recently, the use of pre-trained models has become increasingly popular due to their comparably low complexity and high accuracy on modern hardware^1–6^. In order for such models to perform well, their empirical parameters require fitting to high-quality reference data. Depending on the application, reference data are either experimental, or come from computationally more expensive *ab initio* calculations. Although there are already a handful of large computational data sets covering small organic molecules^7–9^, such data is still scarce for larger periodic systems (*cf*. Materials Cloud Archive^10,11^ or the NOMAD database^12,13^). Motivated by this fact, we present a quantum-chemical data set for zeolites. Zeolites are porous materials comprised of interconnected SiO_4_ or AlO_4_ tetrahedra. Their properties can be fine-tuned through synthesis of materials with specific pore size, or the inclusion of additional metal cation sites^14–17^. Because of their topology and synthetic flexibility, zeolites have various applications as adsorbents^18–20^ and catalysts^17,21–23^. To this day, a myriad of different zeolite framework types is available experimentally, and many more hypothetical structures can be derived^24–26^. The documentation of fundamental zeolite framework types and derived materials has led to the publication of the well-known *Atlas of Zeolite Structures*^27^ in several editions. The atlas lists each unique framework type by its three-letter-code, as assigned by the by the Structure Commission of the International Zeolite Association (IZA). Today, its contents are available online at the *Database of Zeolite Structures*^28^, which we use as a source of initial structures for our data set. In this first installment, we include properties for 204 out of the currently available 256 zeolite framework types in the database (a total of 226 unique geometries when also considering derived materials). Our descriptor provides the complete optimization trajectories for each system with atomic positions, lattice vectors, atomic gradients and stress tensors at each step. We envision future extensions of the data set to focus on derived geometries, covering structural defects and host-guest interactions.

## Methods

Initial zeolite structures are collected from the public *Database of Zeolite Structures*^28^ in the *Crystallographic Information File* (CIF) format, before conversion to the XYZ format with the Atomic Simulation Environment^29^ (ASE) package. After selection of all systems with less than 301 atoms, each is manually filtered by removing redundant atom positions in case of fractional occupancies and adding missing hydrogen atoms where needed. Each structure’s coordinates and cell parameters are energy-minimized with the periodic density functional code BAND^30^, as implemented in the Amsterdam Modeling Suite^31^ (AMS). The calculations are performed with the revPBE functional^32,33^, a ‘Small’ frozen core and the double-*ζ* polarized (DZP) basis set. Grimme’s D3(BJ) dispersion correction^34^ is applied to all calculations. Previous research has shown that the selected level of theory can accurately reproduce zeolite geometries, albeit slightly overestimating the Si-O bond length (in the range of 2 pm) and smaller Si-O-X angles (in the range of 5 degrees) when compared to experimental results^35,36^. At the same time, dispersion-corrected functionals are generally more accurate when describing adsorption processes^37–39^. For the optimization of the initial structures, geometry convergence criteria are left at their default values, namely 0.001 Hartree/Å, 0.00001 Hartree/Atom and 0.1 Å for atomic gradients, energy and atomic displacements respectively. We use a Quasi-Newton optimizer^40^ in the delocalized coordinates space for the initial optimizations. Cases of problematic convergence are restarted with the FIRE^41^ optimizer.

## Data Records

The data is made available at the Materials Cloud Archive^42^. Each system’s trajectory is stored in an individual NumPy^43^. *npz* file. We describe the data types held in each file in Table 1, storing the complete geometry optimization trajectory, including atomic coordinates, system energies, nuclear gradients, lattice vectors and stress tensors for each geometry optimization step. Entries at the first position correspond to the input structure; the last position holds the data for the final, optimized structure. Hirshfeld partial charges^44^ are provided for the final (optimized) geometries. Atomic coordinates and lattice vectors are stored in ångström, all other properties are stored in atomic units.

**Table 1:** Overview of the data structures stored in a .npz file.

Data	Unit	Key	Array Shape
Atomic Numbers	—	numbers	(*R*,)
Atomic Coordinates	Å	xyz	(*N*, *R*, 3)
*x*-, *y*- and, *z*-Components of the Lattice Vectors	Å	lattice	(*N*, 3, 3)
Energy	hartree	energy	(*N*,)
Nuclear Gradients	hartree/bohr	gradients	(*N*, *R*, 3)
Stress Tensors	atomic units	stress	(*N*, 3, 3)
Hirshfeld Charges	atomic units	charges	(*R*,)

Each array can be accessed through the respective key. The variables *N* and *R* denote the number of geometry optimization steps and the system size respectively. Partial charges are only computed for the last geometry.

## Technical Validation

**Fig. 1:**
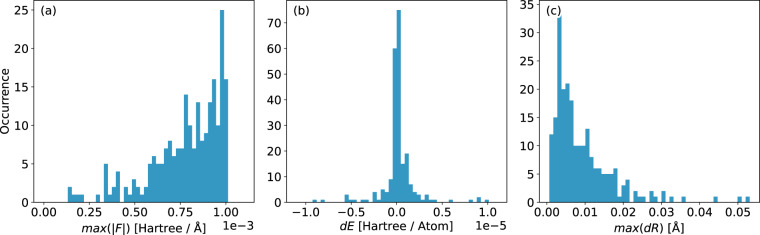
Distribution of convergence criteria at the last optimization step for all calculated systems in the data set. Showing (**a**) the highest absolute component of all nuclear gradients, (**b**) change in system energy and (**c**) highest relative atomic displacement.

**Fig. 2:**
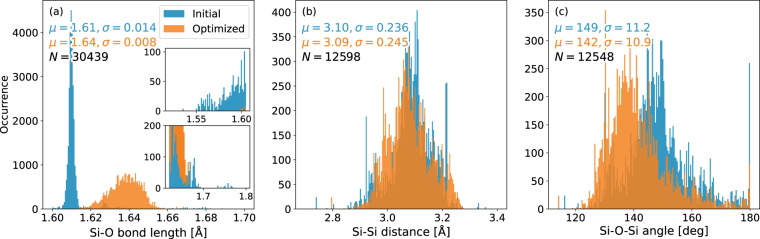
Distributions of (**a**) Si-O bond lengths, (**b**) Si-Si distances in the second coordination sphere and (**b**) Si-O-Si angles as calculated from all geometries in the data set. Blue and orange bars denote data from initial and optimized geometries, respectively. Mean *μ* and standard deviation *σ* printed in the same color as the underlying data. *N* denotes the total sample size.

**Fig. 3:**
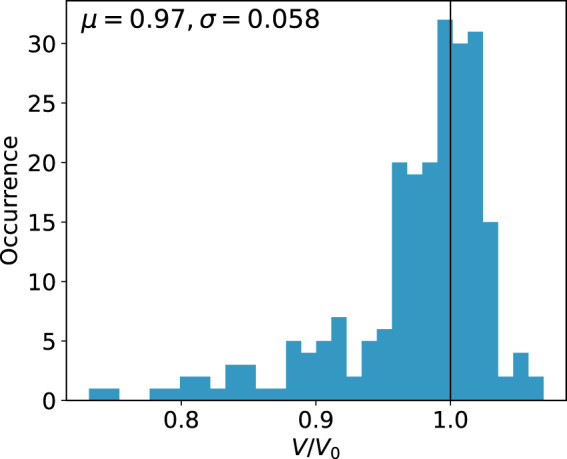
Distribution of relative cell volumes per system as the quotient of optimized-to-initial cell volumes. Values below 1 describe a shrinking cell as the optimization progresses. Black line marks *V*/*V*_0_  =  1. Sample size is 226.

**Fig. 4:**
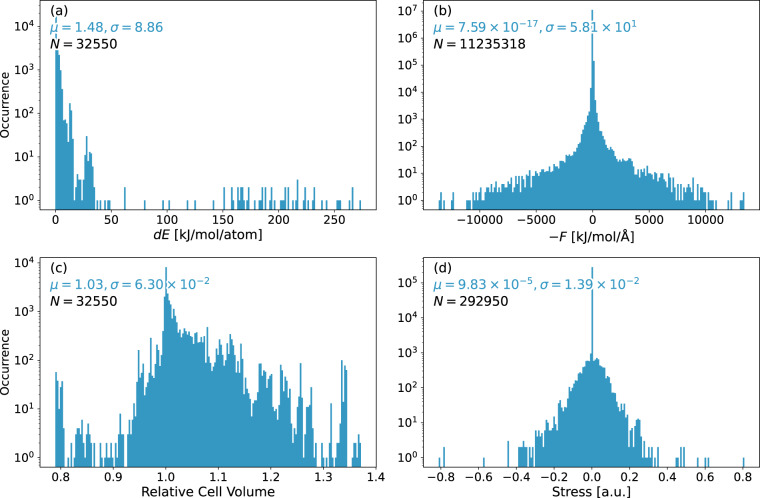
Distributions of physical quantities in the data set. Showing (**a**) energy differences per atom, relative to the respective energy of the optimized system; (**b**) atomic gradient components; (**c**) unit cell volumes, relative to the optimized system’s volume; (**d**) stress tensor components. Data is printed on a logarithmic y-scale for a clear display of the distribution. Mean *μ* and standard deviation *σ* printed in the same units as the underlying data. *N* denotes the total sample size.

The complete data set includes geometry optimizations of 226 systems, resulting in a total of 32550 geometries. System sizes range between 15 and 334 atoms (mean: 126). We illustrate the convergence of all reference calculations in Fig. 1, showing that all optimized systems are well within the defined convergence criteria. Elemental occurrences in the data set are listed in Table 2. Si-O, Si-Si distances as well as Si-O-Si angles are presented in Fig. 2 as the most prominent geometrical descriptors. As most of the initial structures from the IZA database are idealized geometries^45^, a sharp mean for the Si-O bond distance can be observed at roughly 161 pm (Fig. 2a, blue histogram). Long tails in the distribution vanish and the mean is shifted towards approximately 164 pm when considering geometry-optimized structures (Fig. 2a, orange histogram). Considering the Si-O-Si angles, a slight shift towards smaller values is observed (mean of 149 vs. 142 degrees, Fig. 2c). Both effects have been previously reported by Fischer *et al*.^35,36^ and are inherent to the selected level of theory. Distributions of the Si-Si distances in the second coordination sphere do not shift significantly when comparing initial and optimized geometries (Fig. 2b). Relative changes in the cell volumes are presented in Fig. 3 as the ratio of each system’s optimized-to-initial volume. Values below 1 translate to a shrinking unit cell as the optimization progresses. Overall, the geometrical descriptors are in good agreement with experimental data^46–51^. Additional averages for bond distances and angles are summarized in Tables 3, 4 respectively. Distributions of energies, atomic gradients, cell volumes and stress tensors are depicted in Fig. 4. As expected from geometry optimization trajectories, all properties have – with the exception of relative cell volumes – a distinct mean close to zero. Structures close to the initial input geometries contribute to the relatively high standard deviations. Evaluation of the relative cell volumes shows a shifted distribution, with roughly 76% of all structures having a larger volume than their respective optimized geometry. A detailed overview of all calculated structures, sorted by their IZA three-letter-code, the system size and number of iterations is provided in Online Table 1.

**Table 2:** Elemental occurrences in the complete data set.

Element	Occurrence
Si	226
O	226
H	21
Al	12
N	4
Ca	4
Ge	3
Li	2
Na	2
K	2
C	2
F	1
Be	1
Cs	1
Ba	1

Counting all structures containing at least one atom of the listed element. Each element’s isolated atomic energy is listed in hartree.

**Table 3:** Mean atomic bond length distributions and their standard deviations (std. dev.) in in ångström.

Bond	Mean	Std. Dev.	Number of points
Si-O	1.638	0.0085	30439
H-O	0.999	0.1174	266
Al-O	1.763	0.0133	234
Ge-O	1.795	0.0239	202
Na-O	2.473	0.0841	104
C-C	1.540	0.0049	100
C-H	1.100	0.0027	98
K-O	3.175	0.4809	61
Ca-O	2.469	0.0925	57
N-H	1.055	0.0913	50
Si-K	3.945	0.3196	41
Cs-O	3.429	0.2820	28
Li-O	1.970	0.0263	21
Be-O	1.669	0.0152	16
Al-K	3.625	0.1650	14
C-N	1.472	0.0037	10
Ba-O	2.903	0.1261	10

Averaged over all geometry-optimized structures.

**Table 4:** Mean Si-O-R angle distributions and their standard deviations (std. dev.) in degrees.

Angle	Mean	Std. Dev.	Number of points
Si-O-Si	148.7	11.2	12548
Si-O-Al	140.6	8.9	170
Si-O-K	106.8	8.8	81
Si-O-Na	112.8	15.1	64
Si-O-Ge	143.2	12.0	52
Si-O-H	110.7	7.9	40
Si-O-Cs	101.6	6.9	36
Si-O-Ca	118.5	16.6	19
Si-O-Be	129.9	0.2	16
Si-O-Li	112.7	4.2	8
Si-O-Ba	112.6	14.1	5

Averaged over all geometry-optimized structures.

## Usage Notes

No data points were filtered as outliers with regards to the distributions of chemical properties (see. Figure 4). Consecutive structures from the same optimization trajectory will be autocorrelated. The data repository provides an interactive plotting script, displaying the system energy, maximum absolute component of the nuclear gradients and the cell volume at every iteration step for each structure. This requires the Bokeh^52^ (v. 2.3.1) package for Python to be installed. SHA-1 hash sums are provided for each file to guarantee data integrity, as well as an example input script for a calculation with BAND. Naming conventions: Derived materials are referred to by their IZA three-letter-code, *e.g*. H-EU-12 is tabulated as ETL_0. Leading non-alphabetical characters have been removed, *e.g*. *-ITN is tabulated as ITN.

## Data Availability

Downloads of the Atomic Simulation Environment^29^ (v. 3.21.1) and NumPy^43^ (v. 1.20.1) packages for Python are freely available. Amsterdam Modeling Suite^31^ (v. 2020.203, r92091) is a commercial software, for which a free trial may be requested at www.scm.com.

## Online Table

**Online Table 1:** Summary of all calculated systems, sorted by their IZA code. Showing the chemical formula, system size R and number of iterations N.

IZA Code	Chemical Formula	R	N
ABW	Si_8_O_16_	24	102
ABW_0	Si_4_Al_4_O_20_Li_4_H_8_	40	30
ACO	Si_16_O_32_	48	4
AEI	Si_48_O_96_	144	16
AEL	Si_40_O_80_	120	16
AEN	Si_48_O_96_	144	137
AET	Si_72_O_144_	216	188
AFG	Si_48_O_96_	144	248
AFI	Si_24_O_48_	72	369
AFN	Si_32_O_64_	96	72
AFO	Si_40_O_80_	120	19
AFR	Si_32_O_64_	96	283
AFS	Si_56_O_112_	168	20
AFT	Si_72_O_144_	216	471
AFV	Si_30_O_60_	90	14
AFX	Si_48_O_96_	144	29
AFY	Si_16_O_32_	48	42
AHT	Si_24_O_48_	72	263
ANA	Si_48_O_96_	144	7
APC	Si_32_O_64_	96	245
APD	Si_32_O_64_	96	408
AST	Si_40_O_80_	120	178
ASV	Si_20_O_40_	60	22
ATN	Si_16_O_32_	48	168
ATO	Si_36_O_72_	108	100
ATS	Si_24_O_48_	72	299
ATT	Si_12_O_24_	36	63
ATV	Si_24_O_48_	72	401
AVL	Si_42_O_84_	126	54
AWO	Si_48_O_96_	144	21
AWW	Si_24_O_48_	72	11
BCT	Si_8_O_16_	24	5
BEA	Si_64_O_128_	192	61
BEC	Si_32_O_64_	96	395
BIK	Si_12_O_24_	36	106
BIK_0	Si_4_Al_2_O_14_Li_2_H_4_	26	54
BOF	Si_24_O_48_	72	18
BOG	Si_96_O_192_	288	21
BOZ	Si_92_O_184_	276	14
BPH	Si_28_O_56_	84	143
BRE	Si_16_O_32_	48	23
BSV	Si_96_O_192_	288	9
CAN	Si_12_O_24_	36	14
CAS	Si_24_O_48_	72	316
CAS_0	Cs_4_Si_20_Al_4_O_48_	76	325
CDO	Si_36_O_72_	108	987
CFI	Si_32_O_64_	96	339
CGF	Si_36_O_72_	108	30
CGS	Si_32_O_64_	96	307
CHA	Si_36_O_72_	108	20
CHI	Si_28_O_60_H_8_	96	25
CON	Si_56_O_112_	168	37
CSV	Si_20_O_40_	60	342
CZP	Si_24_O_48_	72	14
DAC	Si_24_O_48_	72	293
DFT	Si_8_O_16_	24	10
DOH	Si_34_O_68_	102	359
DON	Si_64_O_128_	192	30
EAB	Si_36_O_72_	108	55
EDI	Si_5_O_10_	15	5
EDI_0	Ba_2_Si_6_Al_4_O_28_H_16_	56	198
EMT	Si_96_O_192_	288	11
EPI	Si_24_O_48_	72	398
ERI	Si_36_O_72_	108	20
ESV	Si_48_O_96_	144	20
ETL_0	Si_72_O_144_	216	11
ETR	Si_48_O_96_	144	10
ETV	Si_14_O_28_	42	25
EWO	Si_24_O_48_	72	21
EWS	Si_96_O_192_	288	19
EZT	Si_48_O_96_	144	12
FAR	Si_84_O_168_	252	39
FER	Si_36_O_72_	108	25
FRA	Si_60_O_120_	180	264
GIS	Si_16_O_32_	48	7
GIU	Si_96_O_192_	288	37
GME	Si_24_O_48_	72	12
GON	Si_32_O_64_	96	16
GOO	Si_32_O_64_	96	1627
GOO_0	Ca_2_Si_12_Al_4_O_42_H_20_	80	195
HEU	Si_36_O_72_	108	46
IFO	Si_32_O_64_	96	179
IFR	Si_32_O_64_	96	15
IFW	Si_64_O_128_	192	138
IFY	Si_48_O_96_	144	13
IRN	Si_92_O_184_	276	417
IRR	Si_52_O_104_	156	14
IRR_0	Ge_12_Si_40_O_104_	156	29
IRY	Si_76_O_154_H_4_	234	241
ISV	Si_64_O_128_	192	40
ITE	Si_64_O_128_	192	27
ITG	Si_56_O_112_	168	29
ITH	Si_56_O_112_	168	65
ITN	Si_54_O_109_H_2_	165	518
ITT	Si_46_O_92_	138	43
ITW	Si_24_O_48_	72	259
IWR	Si_56_O_112_	168	47
JBW	Si_6_O_12_	18	12
JNT	Si_32_O_64_	96	33
JRY	Si_24_O_48_	72	232
JSN	Si_16_O_32_	48	16
JSR	Si_96_O_192_	288	11
JST	Si_48_O_96_	144	8
JSW	Si_48_O_96_	144	19
KFI	Si_96_O_192_	288	170
LAU	Si_24_O_48_	72	15
LAU_0	Ca_4_Si_16_Al_8_O_64_H_32_	124	306
LEV	Si_54_O_108_	162	16
LEV_1	Si_54_O_108_N_8_C_60_H_104_	334	14
LIO	Si_36_O_72_	108	660
LIT	Si_24_O_52_H_8_	84	24
LOS	Si_24_O_48_	72	393
LOV	Si_18_O_36_	54	468
LTA	Si_24_O_48_	72	41
LTJ	Si_16_O_32_	48	11
LTJ_0	Si_8_Al_8_O_36_N_8_H_40_	100	172
LTL	Si_36_O_72_	108	14
MAR	Si_72_O_144_	216	561
MAZ	Si_36_O_72_	108	42
MEI	Si_34_O_68_	102	307
MEL	Si_96_O_192_	288	47
MEP	Si_46_O_92_	138	379
MER	Si_32_O_64_	96	131
MFI	Si_96_O_192_	288	175
MFS	Si_36_O_72_	108	1351
MON	Si_16_O_32_	48	86
MOR	Si_48_O_96_	144	43
MRE	Si_48_O_96_	144	11
MRT	Si_24_O_48_	72	80
MSO	Si_90_O_180_	270	71
MTF	Si_44_O_88_	132	15
MTT_0	Si_24_F_2_O_48_N_2_H_8_	84	298
MTW	Si_28_O_56_	84	386
MVY	Si_12_O_24_	36	122
MWW	Si_72_O_144_	216	16
NAB	Si_10_O_20_	30	455
NAB_0	Si_16_Na_8_O_56_Be_4_H_32_	116	72
NAT	Si_20_O_40_	60	303
NAT_0	Si_24_Al_16_Na_16_O_96_H_32_	184	103
NAT_3	Ca_4_Si_12_Al_8_O_52_H_24_	100	251
NON	Si_88_O_176_	264	7
NPO	Si_6_O_12_	18	19
NPT	Si_36_O_72_	108	9
NSI	Si_12_O_24_	36	73
OBW	Si_76_O_152_	228	11
OFF	Si_18_O_36_	54	37
OKO	Si_68_O_136_	204	35
OSI	Si_32_O_64_	96	11
OSO	Si_9_O_18_	27	8
OWE	Si_16_O_32_	48	39
PAR	Si_32_O_68_H_8_	108	24
PCR	Si_60_O_120_	180	40
PCR_0	Si_60_O_120_	180	38
PCS	Si_64_O_128_	192	32
PHI	Si_32_O_64_	96	882
PON	Si_24_O_48_	72	33
POR	Si_64_O_128_	192	288
POS	Si_64_O_128_	192	21
PTT	Si_24_O_48_	72	355
PTY	Si_10_O_20_	30	20
PUN	Si_36_O_72_	108	30
PWO	Si_20_O_40_	60	307
PWW	Si_40_O_80_	120	30
RHO	Si_48_O_96_	144	7
RRO	Si_18_O_36_	54	35
RSN	Si_36_O_72_	108	17
RTE	Si_24_O_48_	72	22
RTH	Si_32_O_64_	96	205
RUT	Si_36_O_72_	108	49
RWY	Si_48_O_96_	144	19
SAF	Si_64_O_128_	192	16
SAO	Si_56_O_112_	168	121
SAS	Si_32_O_64_	96	338
SAT	Si_72_O_144_	216	23
SAV	Si_48_O_96_	144	730
SBN	Si_10_O_20_	30	14
SBS	Si_96_O_192_	288	51
SEW	Si_66_O_132_	198	32
SFE	Si_14_O_28_	42	178
SFF	Si_32_O_64_	96	256
SFG	Si_74_O_148_	222	212
SFH	Si_64_O_128_	192	26
SFN	Si_32_O_64_	96	313
SFO	Si_32_O_64_	96	19
SFS	Si_56_O_112_	168	26
SIV	Si_64_O_128_	192	49
SOD	Si_12_O_24_	36	5
SOF	Si_40_O_80_	120	372
SOR	Si_48_O_96_	144	18
SOS	Si_24_O_48_	72	323
SSF	Si_54_O_108_	162	145
SSY	Si_28_O_56_	84	335
STF	Si_32_O_64_	96	22
STF_0	Si_16_O_32_	48	48
STI	Si_72_O_144_	216	13
STT	Si_64_O_128_	192	21
STW	Si_60_O_120_	180	40
SVR	Si_92_O_192_H_16_	300	27
SVV	Si_56_O_112_	168	61
SWY	Si_72_O_144_	216	21
SZR	Si_36_O_72_	108	12
SZR_0	K_4_Si_32_Al_4_O_72_	112	32
TER	Si_80_O_160_	240	114
THO	Si_10_O_20_	30	225
TOL	Si_72_O_144_	216	217
TON	Si_24_O_48_	72	334
TON_0	Si_24_O_48_N_1_C_4_H_11_	88	389
UEI	Si_48_O_96_	144	40
UFI	Si_64_O_128_	192	30
UFI_0	K_8_Si_56_Al_8_O_128_	200	243
UOE	Si_12_O_24_	36	13
UOS	Si_24_O_48_	72	11
UOZ	Si_40_O_80_	120	19
USI	Si_40_O_80_	120	31
UTL	Si_76_O_152_	228	70
UTL_0	Ge_16_Si_60_O_152_	228	216
UWY	Si_60_O_120_	180	26
UWY_0	Ge_36_Si_24_O_142_H_44_	246	512
VET	Si_17_O_34_	51	384
VFI	Si_36_O_72_	108	288
VSV	Si_36_O_72_	108	27
WEI	Si_20_O_40_	60	17
WEN	Si_20_O_41_H_2_	63	357
YUG	Si_16_O_32_	48	22
YUG_0	Ca_2_Si_12_Al_4_O_40_H_16_	74	372
ZON	Si_32_O_64_	96	307
